# Interactions between lipids and proteins are critical for organization of plasma membrane-ordered domains in tobacco BY-2 cells

**DOI:** 10.1093/jxb/ery152

**Published:** 2018-05-02

**Authors:** Kevin Grosjean, Christophe Der, Franck Robert, Dominique Thomas, Sébastien Mongrand, Françoise Simon-Plas, Patricia Gerbeau-Pissot

**Affiliations:** 1 Agroécologie, AgroSup Dijon, CNRS, INRA, Université Bourgogne Franche-Comté, Dijon, France; 2 Laboratoire de Biogenèse Membranaire (LBM), Unité Mixte de Recherche UMR, CNRS, Université de Bordeaux, Bordeaux, France

**Keywords:** di-4-ANEPPDHQ, cell wall, cytoskeleton, lipid raft, membrane organization, ordered domains, protein-lipid interactions

## Abstract

The laterally heterogeneous plant plasma membrane (PM) is organized into finely controlled specialized areas that include membrane-ordered domains. Recently, the spatial distribution of such domains within the PM has been identified as playing a key role in cell responses to environmental challenges. To examine membrane order at a local level, BY-2 tobacco suspension cell PMs were labelled with an environment-sensitive probe (di-4-ANEPPDHQ). Four experimental models were compared to identify mechanisms and cell components involved in short-term (1 h) maintenance of the ordered domain organization in steady-state cell PMs: modulation of the cytoskeleton or the cell wall integrity of tobacco BY-2 cells; and formation of giant vesicles using either a lipid mixture of tobacco BY-2 cell PMs or the original lipid and protein combinations of the tobacco BY-2 cell PM. Whilst inhibiting phosphorylation or disrupting either the cytoskeleton or the cell wall had no observable effects, we found that lipids and proteins significantly modified both the abundance and spatial distribution of ordered domains. This indicates the involvement of intrinsic membrane components in the local physical state of the plant PM. Our findings support a major role for the ‘lipid raft’ model, defined as the sterol-dependent ordered assemblies of specific lipids and proteins in plant PM organization.

## Teaser

In contrast to the cytoskeleton and the cell wall, lipids and proteins are the main determinants of steady-state regulation of both the number and local distribution of plasma membrane-ordered domains in tobacco cells.

## Introduction

Plant cells are delimited by a plasma membrane (PM) that protects them against the external environment, and that also regulates what (and how much) enters the cell. The PM defines the boundary between the intracellular and extracellular space and also plays a major role in transducing various signals into the appropriate adaptive responses. Since the advent of the classic ‘Fluid Mosaic Model’, consisting of a homogeneous lipid bilayer with embedded proteins arranged as mosaic-like structures (Singer and Nicolson, 1972), multiple lines of evidence have suggested the presence of a nanoscale lateral heterogeneity with regards to the composition and biophysical properties of plant PMs.

One of the original concepts developed within this framework is the ‘lipid raft’ model (Simons and Ikonen, 1997; Simons and Gerl, 2010; Nicolson, 2014), which is based on the sub-division of membranes into regions further defined as ‘small (10–200 nm), heterogeneous, highly dynamic, sterol- and sphingolipid-enriched domains’ (Pike, 2006). According to this model, preferential interactions between cholesterol and sphingolipids generate liquid-ordered (Lo) phase separation (Phillips *et al.*, 1970; Shimshick and McConnell, 1973; Grant *et al.*, 1974; Lentz *et al.*, 1976), in which the resulting membrane domains are highly ordered and tightly packed relative to the surrounding regions (Simons and Sampaio, 2011). The similar ability of plant-specific sterols to form an Lo phase has been reported in PM-purified fractions (Roche *et al.*, 2008) as well as in living tobacco cells (Gerbeau-Pissot *et al.*, 2014), with diffing capabilities to stabilize the lipid bilayer depending on the phytosterol structure (Rujanavech *et al.*, 1986; Schuler *et al*., 1990, 1991; Hartmann, 1998; Halling and Slotte, 2004). This capacity of phytosterols to modulate the size and proportion of the Lo phase in the model membrane has been associated with their ability to interact with plant sphingolipids (Grosjean *et al.*, 2015).

Enrichment in sterols and sphingolipids has been identified for detergent-resistant membrane (DRM) fractions from the PMs of tobacco (Mongrand *et al.*, 2004; Moscatelli *et al.*, 2015), *Arabidopsis thaliana* (Borner *et al.*, 2005; Minami *et al.*, 2009), *Medicago truncatula* (Lefebvre *et al.*, 2007), leek (Laloi *et al.*, 2007), maize and bean (Carmona-Salazar *et al.*, 2011), confirming their association within specific membrane regions. In the tobacco PM, use of immunogold electron microscopy has revealed localization of glycosyl inositol phosphorylceramide (GIPC), the major class of sphingolipids in plants, within 35-nm diameter domains (Cacas *et al.*, 2016). Phosphatidylinositol 4,5-bisphosphate (PIP2) is similarly found in ~25-nm diameter clusters (Furt *et al.*, 2010), suggesting that there is a heterogeneous spatial distribution of the lipids on a nanometre scale. Furthermore, various proteins have also shown a grouped distribution (about 70 nm in size) in tobacco leaf (Raffaele *et al.*, 2009), tobacco BY-2 suspension (Noirot *et al.*, 2014), and Arabidopsis (Li *et al.*, 2012) cells. Plant cell PMs are thus covered with different types of domains enriched in specific membrane-resident proteins (Jarsch *et al.*, 2014). Interestingly, the integrity of some of these protein clusters is sensitive to the amount of sterol, as observed after methyl-β-cyclodextrin (MβCD)-induced sterol depletion (Vereb *et al.*, 2000; Raffaele *et al.*, 2009), which suggests sterol-driven formation of these protein-enriched domains. Furthermore, a close relationship between the sterol amount and polar localization of auxin efflux carriers has been proposed, since sterol methyltransferase 1 (SMT1) and sterol-dependent endosomal recycling are essential in maintaining the polar localization of PIN1 and PIN3 (Willemsen *et al.*, 2003), in addition to PIN2 (Men *et al.*, 2008; Titapiwatanakun and Murphy, 2009).

While lipid-driven segregation can be coupled with additional actin cytoskeleton-based processes to spatially organize the dynamic status of membrane proteins in animal cells (Lenne *et al.*, 2006), no similar actin-dependent mechanisms have yet been fully described in plant cells. Nevertheless, interactions and crosstalk between microtubules and microfilaments have been reported in plant cells (Petrásek and Schwarzerova, 2009), revealing the role of microtubule organization in modulating plant protein motility (Szymanski *et al.*, 2015; Lv *et al.*, 2017). Furthermore, the pattern of cellulose deposition in the cell wall has been shown to strongly affect the trajectory and speed of PM protein diffusion in *Nicotiana tabacum* leaves (Martinière *et al.*, 2012), suggesting the involvement of the cell wall in regulating plant PM lateral organization.

**Fig. 1.**
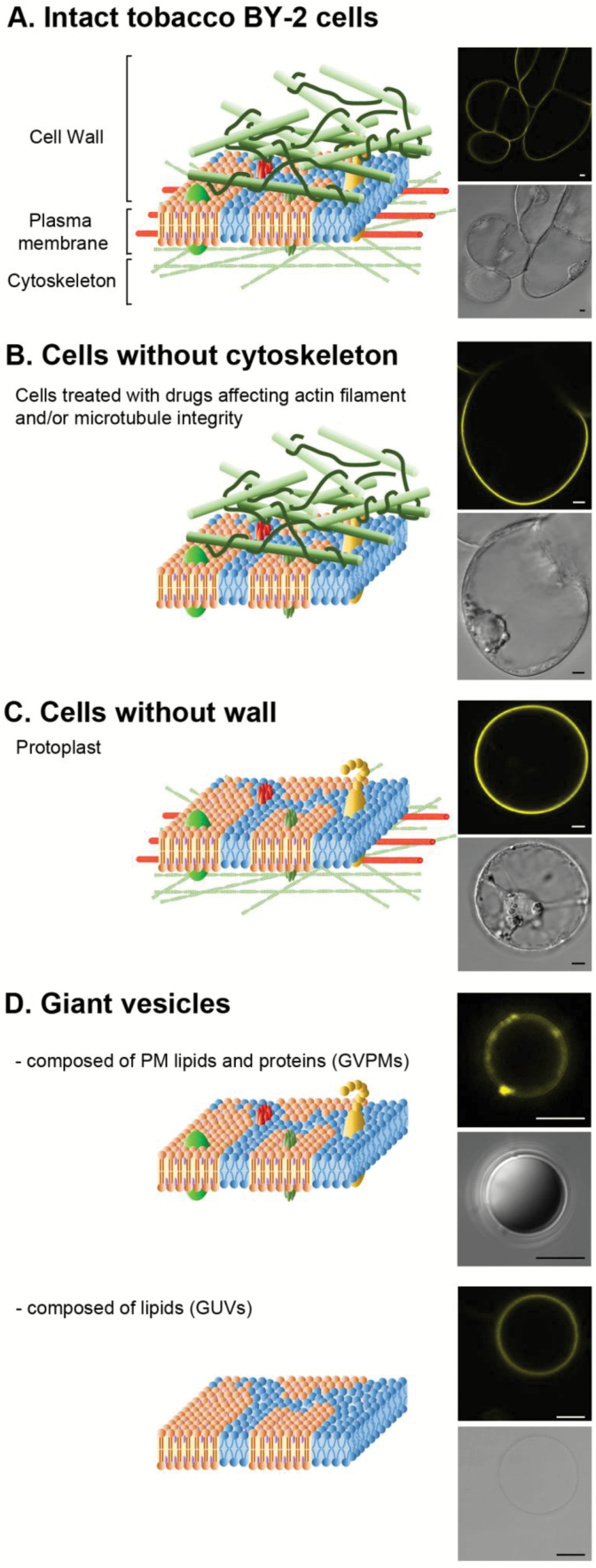
Different models used to characterize the cellular determinants of plasma membrane order. (A) Intact whole BY-2 suspension cells in which the plasma membrane, composed of ordered domains (in orange), is tightly connected with the underlying filaments of the cytoskeleton network and/or the surrounding fibres of the cell wall meshwork. (B) Treatment of BY-2 suspension cells with chemicals disrupts the cytoskeletal components. (C) Protoplasts obtained by enzymatic digestion of BY-2 suspension cells. (D) Giant vesicles were either formed from the whole diversity of tobacco PM lipids (giant unilamellar vesicles, GUVs) or from the direct electrofusion of purified PM vesicles, thus containing both proteins and lipids in their original amounts (giant vesicles of native plasma membrane, GVPMs). Samples were observed by microscopy, using either differential interference contrast (DIC; in grey-scale), or fluorescence after di-4-ANEPPDHQ labelling (excitation at 488 nm; emission acquired in a 520–680 nm band-pass, in yellow). Scale bars are 5 µm.

These previous reports indicate that the plant PM consists of heterogeneously distributed components, in which grouped localization has been demonstrated for some lipid raft markers, including proteins and lipids. However, few studies have characterized the spatial distribution of these clusters at the whole-cell scale. Nonetheless, these data have independently described specific ways that, either alone or in combination, can account for PM spatial organization in plant cells (Ovecka *et al.*, 2010; Li *et al.*, 2012; Gerbeau-Pissot *et al.*, 2014; Sandor *et al.*, 2016), and they justify a comprehensive analysis of the global mechanisms responsible for the distribution of ordered domains. In order to obtain a snapshot evaluation of the respective influence of each of these different molecular and cellular elements on the features of PM lateral organization, we have compared the biophysical properties of several experimental systems with decreasing complexity, all of which correspond to tobacco BY-2 cells in a resting state. We used di-4-ANEPPDHQ, a lipid packing-sensitive dye capable of assessing different levels of membrane order, coupled with confocal microscopy. This approach allowed us to measure the global level of membrane order of the entire PM together with the spatial distribution of ordered domains in either intact tobacco cells, cells with a disrupted cytoskeleton, or cells devoid of a cell wall. The same parameters were also measured in model membranes including giant unilamellar vesicles (GUVs) composed of lipids extracted from the tobacco PM, and giant vesicles of native PMs (GVPMs) from tobacco cells (see Fig. 1 for rationale). Our comprehensive comparisons of the individual ability of membrane and cell elements to modify membrane order revealed a major role for lipids in promoting the formation of ordered domains, and the capacity of proteins to limit this formation. Our results suggest that PM components appear to act together to regulate plant PM heterogeneity, whilst neither the cytoskeleton nor the cell wall seem to play a significant role in the short-term control of ordered domain distribution within steady-state cell PMs. Phosphorylation events also failed to regulate PM organization of tobacco cells in a resting state. Finally, we discuss how our results may support the ‘lipid raft’ model in our understanding of the mechanisms that control the spatial distribution of ordered domains within the plant PM.

## Materials and methods

### Cell growth conditions

Wild-type BY-2 (*Nicotiana tabacum* cv. Bright Yellow-2) cells were grown in Murashige and Skoog (MS) modified medium (basal salt mixture, M0221, Duchefa) at pH 5.6, supplemented with 1 mg l^–1^ thiamine-HCl, 0.2 mg l^–1^ 2,4 dichlorophenylacetic acid, 100 mg l^–1^ myo-inositol, 30 g l^–1^ sucrose, 200 mg l^–1^ KH_2_PO_4_, and 2 g l^–1^ MES. Cell suspensions were maintained under continuous light conditions (200 µE m^–2^ s^–1^) on a rotary shaker (140 rpm) and diluted (4:80) weekly into fresh medium.

### Chemicals treatments

BY-2 cells were equilibrated according to Gerbeau-Pissot *et al.* (2014). After a 2-h cell incubation period, concentrated stock solutions (1000× in DMSO) of the cytoskeleton inhibitors cytochalasin D, latrunculin B, nocodazole, and oryzalin (Sigma-Aldrich), were individually added to cell suspensions at a final concentration of 50 µM, 10 µM, 20 µM, and 10 µM, respectively. Control cells were incubated with the same dilution of DMSO. Cells were treated for 1 h on a rotary shaker (120 rpm) at 25 °C before observation. Cells were subsequently plasmolysed in I2 (0.5 mM CaCl_2_, 0.5 mM K_2_SO_4_, and 2 mM MES, pH 5.9) containing 400 mM mannitol (instead of 175 mM used by Gerbeau-Pissot *et al.*, 2014) for several minutes before observation. Staurosporine (Sigma-Aldrich) was added to the cell suspension from concentrated stock solutions in DMSO, taking care not to exceed the final DMSO concentration (0.5 % v/v).

### Protoplast preparation and cell wall regeneration

The protoplast preparation protocol was adapted from (Zaban *et al.*, 2013). All steps were performed in sterile conditions. The BY-2 cells were collected and centrifuged at 100 *g*. Cells were washed in 0.4 M mannitol at pH 5.5 and centrifuged again, then resuspended in an enzymatic solution (Pectolyase Y23 0.1 % w/v, cellulose Onozuka RS 1 % w/v in 0.4 M mannitol at pH 5.5) and digested for 4–5 h at 25 °C, under shaking at 65 rpm in Petri dishes. Protoplasts were harvested after centrifugation (500 *g* for 5 min) and washed three times in FMS wash medium (4.3 g l^–1^ MS salts, 100 mg l^–1^ myo-inositol, 0.5 mg l^–1^ nicotinic acid, 0.5 mg l^–1^ pyridoxine-HCl, 0.1 mg l^–1^ thiamine, 10 g l^–1^ sucrose in 0.25 M mannitol, pH 5.8). For cell wall regeneration, protoplasts were transferred to FMS-store medium (FMS with 0.1 mg l^–1^ 1-naphthaleneacetic acid and 1 mg ml^–1^ benzylaminopurin) and incubated at 26 °C in the dark, with shaking in Petri dishes. Protoplasts were observed at 0, 24 h, 48 h, and 5 d after digestion.

### Preparation of GUVs

Giant unilamellar vesicles (larger than 10µm) were prepared as follows.

#### Tobacco PM isolation

PM fractions were obtained from BY-2 cells by membrane partitioning in an aqueous polymer two-phase system with polyethylene glycol 3350/dextran T-500 (6.6% each), according to Mongrand *et al.* (2004). Protein content was quantified using the Bradford method, in order to obtain an aliquoted solution of 10 mg ml^–1^ final concentration.

#### Purification and quantification of tobacco PM lipids

Polar lipids were extracted according to three independent methods detailed in Cacas *et al.* (2016) and based on different extraction solvent mixtures, namely chloroform/methanol/HCl (200/100/1, v/v/v), methyl *tert*-butyl ether (MTBE)/methanol/water (100/30/25, v/v/v), or a lower phase of propan-2-ol/hexane/water (55/20/25, v/v/v). GIPCs were purified according to a method adapted from Carter and Koob (1969) to obtain sufficient amounts for GUV production and lipid quantification, as described in Buré *et al.* (2011). Extracted lipids were dissolved in chloroform/methanol/water (30/60/8, v/v/v) for storage and further quantified by GC-MS according to Buré *et al.* (2011).

#### GUV production

GUVs were prepared by electroformation in a flow chamber (ICP-25 Perfusion Imaging Chamber, Dagan) connected to a function generator (PCGU1000, Velleman) and a temperature controller (TC-10, Dagan). Tobacco PM fractions (2 µg of proteins) or a mixture of tobacco PM lipids corresponding to a final phospholipid/sphingolipid/sterol composition of 4/4/1.5 (w/w/w, 2 µg final) were deposited on two microscope slides (18 × 18 mm) coated with electrically conductive indium tin oxide (resistivity 8–12 ohms). Lipid-coated slides were placed under a vacuum and away from light for at least 2 h until a thin film was obtained. Cover slips were set up in the flow chamber, and the lipid layer was rehydrated with 200 µl of swelling solution (25 mM HEPES, 10 mM NaCl, and 100 mM sucrose) pre-heated to 40 °C for lipid GUVs. A voltage of 3.5 V (adjustable during the experiment) at 10 Hz and a temperature of 40 °C were applied for a 2-h minimum period in a light-protected environment. After lipid swelling, the temperature of the chamber was slowly cooled to 22 °C (2 h minimum cooling time).

### Fluorescence labelling

To examine cytoskeleton integrity, rhodamine-phalloidin (Invitrogen, 0.1 mg ml^–1^, 30 min) and Tubulin Tracker^TM^ (Invitrogen, 50 µM, 45 min) were used to detect actin filaments and microtubules, respectively. To determine whether the cell wall was present samples were examined after staining with calcofluor-white (Sigma-Aldrich, 0.01 %, w/v) for several minutes. The resulting fluorescence signal of this product reveals cellulose and chitin structures. To determine the membrane order, GUV suspensions or tobacco cells were labelled with the fluorescent probe 1-[2-Hydroxy-3-(N,N-di-methyl-N-hydroxyethyl)ammoniopropyl]-4-[β-[2-(di-n-butylamino)-6-napthyl]vinyl] pyridinium dibromide (di-4-ANEPPDHQ; Invitrogen, stock solution in DMSO, 3 µM final, 1–2 min).

### Confocal fluorescence microscopy

Labelled cells were deposited between the slide and the cover slip and observed with a Leica TCS SP2-AOBS laser scanning microscope (Leica Microsystems) coupled to a HCPL Apochromat CS 63× (N.A. 1.40) oil immersion objective. Fluorescence excitations were obtained using either the 543-nm line of a helium-neon laser (rhodamine-phalloidin), the 488-nm line of an argon laser (tubulin tracker), or a 405-nm diode (calcofluor). Fluorescence emissions were recorded between 555–700 nm (rhodamine-phalloidin), 500–600 nm (tubulin tracker), and 410–480 nm (calcofluor). For di-4-ANEPPDHQ observation, after excitation at 488 nm, emission intensities were acquired between 540–560 nm (green image) and between 650–670 nm (red image). Ratiometric imaging was performed using the ImageJ software (http://imagej.nih.gov/ij/).

### Fluorescence spectroscopy

Sample solutions (1 ml) were placed in a 10-mm special optic path glass cuvette filled in a thermoelectric cooler (24 °C, Wavelength Electronics, Inc.). Fluorescence measurements were performed using a Fluorolog-3 FL3-211 spectrometer (Jobin-Yvon, Horiba Group). The emission spectrum was monitored by one photomultiplier (520–700 nm). A xenon arc lamp (488 nm) was used as the light source. Data acquisitions were performed using the Datamax software (Jobin-Yvon/Thermo Galactic, Inc.).

### Statistical tests

Statistical analyses were based on non-parametric tests (Mann–Whitney), since we observed that our data exhibited a non-Gaussian distribution.

## Results

### Phosphorylation does not regulate the level of PM order or the distribution of ordered domains

We previously described the heterogeneity of tobacco PM lateral organization with respect to local membrane order by measuring the distribution of the liquid-ordered/liquid-disordered (Lo/Ld) phases (Gerbeau-Pissot *et al.*, 2014). In order to identify key players that govern this PM lateral organization, we exploited the fluorescence properties of the environment-sensitive dye di-4-ANEPPDHQ (Jin *et al.*, 2005), the red/green ratio of which is inversely correlated with the level of membrane order (Jin *et al.*, 2006). BY-2 suspension cells were stained for 2 min with di-4-ANEPPDHQ (3 µM) and observed by confocal microscopy. We then measured the fluorescence intensities of the entire PM and evaluated the membrane order using the RGM parameter (red-to-green ratio of the membrane, RGM=*I*_660_/*I*_550_). To characterize the spatial distribution of PM ordered domains, we calculated the RGM value of the PM in tobacco cell regions, hereafter referred to as RGR for ‘red-to-green ratio of the ROI’, where ROI is a ‘region of interest’ corresponding to a 300 × 300 nm square.

**Fig. 2.**
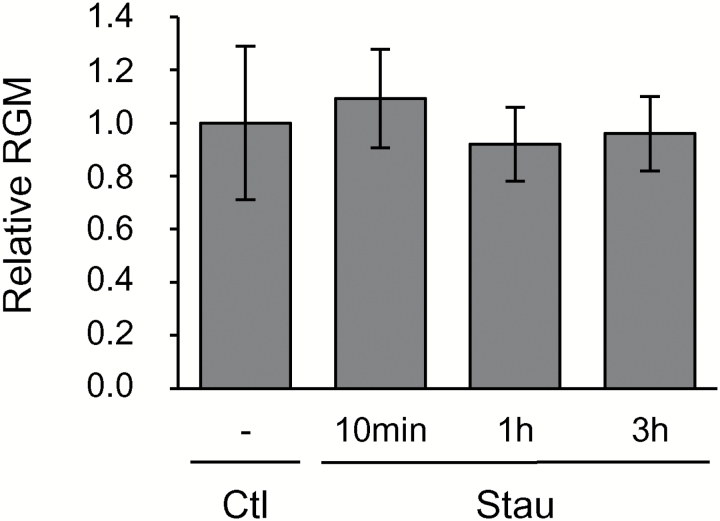
Effect of a protein kinase inhibitor on membrane order of living tobacco cells. After incubation (for 10 min, 1 h, or 3 h) with the kinase inhibitor staurosporine (Stau; 2.5 µM), cells were labelled for 2 min with di-4-ANEPPDHQ (3 µM). Cells were subsequently analysed by spectrofluorimetry, and membrane order was quantified using the red-to-green membrane fluorescence ratio (RGM, =*I*_660_/*I*_550_). The RGM of PMs from treated cells is shown as a relative value compared to control cells (Ctl, with equivalent volume of DMSO). Data are means (±SD), *n*=26 independent experiments.

To test for the contribution of active pathways in the control of these parameters, tobacco suspension cells were treated with staurosporine, a broad-spectrum protein-serine/threonine kinase inhibitor (Suzuki and Shinshi, 1995). Addition of staurosporine (2.5 µM) failed to modify the RGM, regardless of the incubation time (from 10 min to 3 h, Fig. 2). In contrast, we were able to determine a significant modification in membrane order using the same experimental set-up under conditions known to induce an increase PM order (Supplementary Fig. S1 at *JXB* online). This indicates that the technique is sensitive enough to detect minor variations, and that the conditions are sufficient to inhibit signalling events (Bonneau *et al.*, 2010; Sandor *et al.*, 2016), demonstrating that the maintenance of membrane order is independent of phosphorylation events. These results suggested that the signalling pathway was not involved in the control of PM order during the course of the experiment. The results prompted us to address the issue of short-term regulation of ordered domain organization via other mechanisms, especially the contribution of physical interactions.

### Cytoskeleton remodelling does not modify the organization of PM-ordered domains

**Fig. 3.**
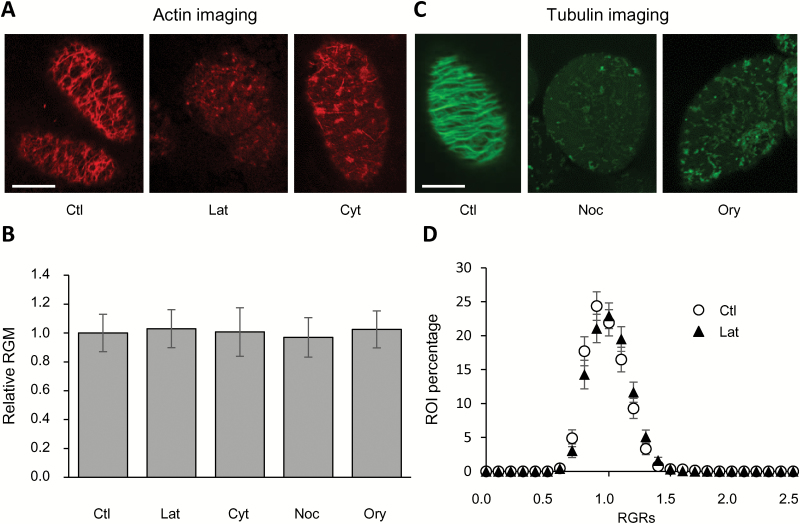
Influence of the cytoskeleton on the membrane order of tobacco cell PMs. (A, C) Effect of pharmacological treatments on cytoskeleton integrity. BY-2 cells were incubated for 1 h with either latrunculin B (Lat, 10 µM), cytochalasin D (Cyt, 50 µM), nocodazole (Noc, 20 µM), or oryzalin (Ory, 10 µM), or with the same concentration of DMSO (control, Ctl). The cytoskeleton was observed by fluorescence microscopy using (A) rhodamine-phalloidin for actin colouration (0.1 mg ml^–1^) and (C) tubulin tracker for microtubule staining (50 µM). After 1 h, patches were detected on treated cells, in comparison to the intact network of filaments observed on control cells. Scale bars are 20 µm. (B) Effect of cytoskeleton integrity on global membrane order. BY-2 cells were exposed to pharmacological treatments that disrupted the actin or tubulin meshwork (as detailed above), or both (Lat, 10 µM and Ory, 10 µM). After 1 h of incubation, cells were labelled for 2 min with di-4-ANEPPDHQ (3 µM). Cells were then observed by confocal microscopy, and membrane order was quantified using the red-to-green membrane fluorescence ratio (RGM, =*I*_660_/*I*_550_). The RGM values obtained for treated cells are shown relative to the values of untreated cells (Ctl, in DMSO). Data are means (SEM), *n*=96–428 from at least five independent experiments. (D) Effect of cytoskeleton integrity on the organization of ordered domains. The distribution of red-to-green membrane fluorescence values (RGR) of individual regions of interest (ROIs, 300 × 300-nm squares) of the PM (control conditions, Ctl) is not influenced by latrunculin B, 10 µM, 1 h). The *x*-axis corresponds to the class of RGR values, and only the maximal value of each class is indicated on the graph. The *y*-axis corresponds to the ROI percentage of each class. Data are means (±SEM), *n*=111–428 cells from at least five independent experiments.

To analyse the potential relationship between the cytoskeleton and BY-2 cell PM order, we measured the evolution of PM order in response to different compounds that affect cytoskeleton components. Brief incubations were performed to exclude any long-term metabolic regulation. Latrunculin B (Spector *et al.*, 1983) and cytochalasin D (Cooper, 1987) bind to actin monomers and prevent their polymerization; their application led to the disruption of actin filaments (Fig. 3A), without any effect on plant cell viability during the 1-h experiment (Supplementary Fig. S2). Monitoring single cells by confocal microscopy showed that these treatments failed to significantly change BY-2 cell RGM (Fig. 3B). No significant differences in RGM were observed between control cells and cells treated with nocodazole (Samson *et al.*, 1979) or oryzalin (Morejohn and Fosket, 1984) (Fig. 3B), both of which can interfere with microtubule polymerization and cause disturbance of the cytoskeleton (Fig. 3C) without affecting cell viability (Supplementary Fig. S2). Spectrofluorimetry measurements, which can assess PM order in batches of thousands of live tobacco cells, confirmed that PM order was independent of actin filament or microtubule polymerization (Supplementary Fig. S3). Simultaneous addition of latrunculin B and oryzalin also did not result in any significant differences between the RGM of control and treated cells (Supplementary Fig. S3), indicating the independence of this parameter with regards to cytoskeleton integrity in living tobacco cells.

**Fig. 4.**
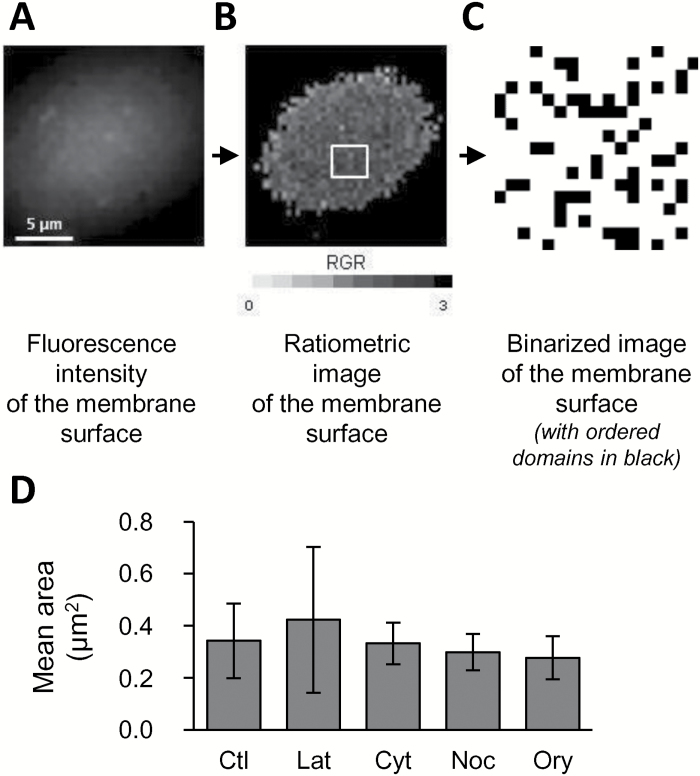
Influence of the cytoskeleton on the spatial distribution of ordered domains. (A) Observation of di-4-ANEPPDHQ-labelled membrane surfaces (excitation at 488 nm; emission corresponds to a 520–680 nm band-pass) with the grey-scale representing fluorescence intensity. (B) The subsequent ratiometric image describing the red-to-green fluorescence ratio of the PM region of interest (RGR) of di-4-ANEPPDHQ within 300 × 300-nm membrane areas displayed in a grey-scale colour-coded representation. (C) A binarized image, representing a detail extracted from the membrane surface focused on the most ordered domains. Recorded regions of interest (ROIs) exhibiting an RGR value within the first quartile of lower RGR values (the most ordered ones) are represented as black pixels. (D) A granulometric approach was then used to compare the spatial distribution of ordered domains on the membrane surface under the different experimental conditions. Tobacco suspension cells were treated for 1 h with a cytoskeletal-active compound (latrunculin B, Lat, 10 µM; cytochalasin D, Cyt, 50 µM; nocodazole, Noc, 20 µM; or oryzalin, Ory, 10 µM) or a control (Ctl) before labelling with di-4-ANEPPDHQ (3 µM, 2 min). The mean area of the black ROI groups is shown. Data are means (±SD), *n*>27 cells from five independent experiments. No significant differences were observed (*P*>0.05).

However, modifications to the lateral organization of membrane order could occur at the nanometre scale without affecting the global RGM. Indeed, the RGM represents the mean value of a multitude of small areas (ROIs) that exhibit different levels of local membrane order (RGR values): the distribution of individual values may be different even though the overall average remains the same. To test this possibility, the emission signals of fluorescently labelled cells were acquired on a tangential plane corresponding to membrane surfaces of 100–500 µm^2^, and the relative abundance of Lo/Ld phases was evaluated. We did not observe any significant difference in the distribution of RGR values between control and latrunculin B-treated cells (Fig. 3D). Furthermore, all of the microtubule- and actin-depolymerizing agents that we tested failed to modify the distribution of RGR values (Supplementary Fig. S4). A granulometric analysis characterizing the aggregation of the most ordered domains was then performed, with a focus on ROIs exhibiting a value in the first quartile of RGR values (Fig. 4–C). This approach did not reveal any significant difference between the size of ordered domains in cells treated with cytoskeletal disruptors and the control cells (Fig. 4D). Taken together, our data suggest that cytoskeleton integrity has no short-term effect on the level of membrane order, either globally (across the entire PM surface) or locally (within small areas at our scale of observation).

### The cell wall does not affect the organization of PM-ordered domains

**Fig. 5.**
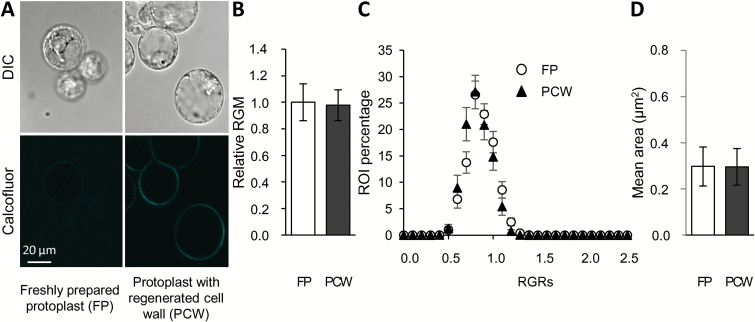
Influence of the cell wall on PM order of BY-2 protoplasts. (A) Cell morphology (differential interference contrast, DIC) and cellulose deposition (fluorescence imaging after calcofluor-white coloration) were analysed for protoplasts, either freshly prepared (3 h) or after cell wall regeneration (1 d after protoplast preparation). (B–D) Confocal microscopy was used to characterize di-4-ANEPPDHQ-labelled protoplasts (3 µM, 2 min), either freshly prepared (FP) or after cell wall regeneration (PCW). (B) Global membrane order was quantified using the red-to-green membrane fluorescence ratio (RGM, =*I*_660_/*I*_550_). (C) Local membrane order was estimated in each region of interest (ROI) of the fluorescent PM, and the distribution of RGM values of individual ROIs of corresponding PMs (RGR) is shown. The *x*-axis corresponds to the class of RGR values, and only the maximal value of each class is indicated on the graph. The *y*-axis corresponds to the ROI percentage of each class. (D) The size of ordered domains was measured as the mean area of groups of pixels corresponding to ROIs exhibiting an RGR value belonging to the first quartile of lower values. Data are means (±SD), *n*>66 cells from five independent experiments.

To investigate the influence of the cell wall on PM order, we compared the RGM of freshly prepared protoplasts devoid of cell walls (1–3 h after enzymatic digestion) and after 24 h of cell wall regeneration (Fig. 5A). The presence of a newly synthesized cell wall, visualized by staining with calcofluor-white (Fig. 5A), did not modify the RGM value (Fig. 5B), suggesting cellulose deposition has no role in the control of PM order. Correspondingly, no modifications in RGM were measured between control and plasmolysed cells when plasmolysis was induced and protoplast shrinkage kept the PM away from the cell wall (Supplementary Fig. S5), suggesting no role in membrane packing for the bounded regions between the cell wall and the PM. Furthermore, the absence of a direct effect of the cell wall suggests that there is no direct contribution from cell wall–PM connections on the regulation of PM order.

A characterization of the abundance of ordered domains was then performed, although the spherical shape of protoplasts limited the size of the tangential area that could be analysed (10–100 µm^2^). No significant difference was observed between the freshly prepared protoplasts and protoplasts in which the cell wall had been regenerated (Fig. 5C), with both conditions displaying the same distribution of RGR values. Live ratiometric imaging also indicated a similar size for ordered domains in protoplasts, before and after cell wall regeneration (Fig. 5D). Thus, the cell wall does not seem to influence the spatial distribution of ordered domains within the tobacco PM.

### Production of giant vesicles from tobacco PMs reveals the involvement of lipids and proteins in the control of the spatial distribution of ordered domains

The data presented above highlight the possibility of a crucial role for intrinsic PM components with regards to PM-ordered domains. To assess the involvement of a wide diversity of PM lipids in the regulation of membrane order, we prepared giant unilamellar vesicles (GUVs) using a mixture of the different classes of lipids in their relative proportions as found in native BY-2 cell PMs. A careful lipidomic analysis highlighted the large diversity of BY-2 PMs (Supplementary Fig. S6), with a phospholipid/sphingolipid/sterol ratio of 4/4/1.5 (w/w/w). The RGM value of these GUVs labelled with di-4-ANEPPDHQ was 0.89 ± 0.16 (±SD, *n*=29). Taking into account the vast array of lipids that comprise PMs, we then characterized the ability of fatty acid saturation to increase membrane order. The results showed that RGM significantly decreased from 2.47 ± 0.24 (*n*=38) to 1.49 ± 0.16 (*n*=20) for GUVs consisting of only 1,2 dioleoylphosphatidylcholine (DOPC) or DOPC/DPPC (DPPC: 1,2 dipalmitylphosphatidylcholine; 1/1, mol/mol), respectively (Supplementary Fig. S7). The complexity level of different lipid combinations (in terms of both number and composition of the different lipid families) similarly modified the GUV membrane order (Supplementary Fig. S7). Overall, this suggests that the diversity of lipid molecules represented in BY-2 cell PMs, together with their specific ability to organize the membrane (Grosjean *et al.*, 2015), could be responsible for the high membrane order reported here for GUVs composed of tobacco PM lipids.

In addition to lipid–lipid interactions, protein–lipid interactions are essential contributors to PM organization (van den Bogaart *et al.*, 2011). Hence it is of primary interest to compare the characteristics of GUVs composed of tobacco PM lipids (as detailed above) with GUVs containing lipids and proteins from tobacco PMs in their native amounts. However, the molecular mechanisms involved in PM vesicle fusion induced by detergents cause significant artefacts, e.g. bilayer–micelle transition (Alonso *et al.*, 1982). Furthermore, technical limitations restrict the protein levels included in these proteoliposomes to 5% (Kahya *et al.*, 2005), far below the level of native PMs, which is estimated to be 50% by weight; consequently, the use of these approaches is restricted. Giant PM vesicles (GPMVs), corresponding to PM blebs detached from cells, have a protein and lipid diversity mirroring the native PM (Scott, 1976) and separate into co-existing Lo/Ld phases, which enables the investigation of the structural determinants of ordered domain association (Baumgart *et al.*, 2007; Kaiser *et al.*, 2009; Levental *et al.*, 2011). However, the cell wall surrounding the plant cell prevents the use of this procedure for tobacco cells. We therefore developed a new protocol to produce giant vesicles of native PMs (GVPMs) by electrofusing small vesicles of purified PM fractions. However, GUV formation using purified PM fractions was extremely difficult due to the presence of proteins and the obstacle they presented to fusion, and so the electroformation method was modified by varying time, temperature, osmolarity, voltage, and frequency in order to enable a high yield of GVPM formation (Supplementary Fig. S8A). By comparing the different protocols, we were able to efficiently produce GVPMs (10^3^ vesicles starting from 2 µg lipids) with a size amenable to observation by confocal microscopy (Supplementary Fig. S8B). Under optimized conditions, 20% of the GVPM population had a diameter larger than 15 µm. The procedure (described in detail the Methods section) can now be used routinely to efficiently prepare GVPMs directly from purified PMs.

**Fig. 6.**
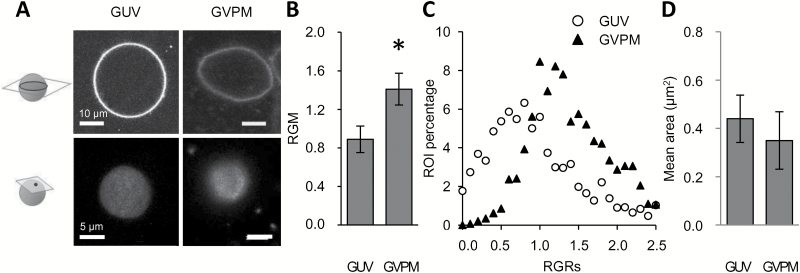
Influence of lipids and proteins on the global and local order of tobacco PMs. Giant unilamellar vesicles (GUVs) were produced by mixing extracts of purified lipid classes according to their relative amounts within the membrane (phospholipids/phytosphingolipids/phytosterols: 4/4/1.5 w/w/w) or by fusing isolated PM vesicles (giant vesicles of native plasma membrane, GVPMs). Vesicles were labelled with di-4-ANEPPDHQ (3 µM, 2 min). (A) Transverse (top) and tangential (below) views of fluorescent vesicles were made using confocal microscopy (excitation at 488 nm; emission corresponds to the sum of fluorescence intensities acquired in a 520–680 nm band-pass) of GUVs and GVPMs. (B) The red-to-green fluorescence ratio (RGM, =*I*_660_/*I*_550_) of fluorescent vesicles was measured using confocal microscopy (dividing the red, 545–565 nm, by the green, 635–655 nm, emission band-passes). Data are means (±SD), *n*>24 experiments; significant difference: *P<0.05. (C) Individual regions of interest (ROIs, 300 × 300 nm) of the surface of fluorescent vesicles were classified according to their RGM values (i.e. giving RGR values). The RGR distribution of a representative GUV composed of a PM lipid mixture is compared to the distribution of GVPMs. The *x*-axis represents the class of RGR values; only the maximal value of each class is indicated on the graph. The *y*-axis represents the percentage of each class of ROI values. (D) The size of ordered domains was measured as the mean area of groups of pixels corresponding to ROIs exhibiting an RGR value belonging to the first quartile of lower values, and is compared for giant vesicles composed of either PM lipids (GUV) or PM lipids and proteins (GVPM). Data are means (±SD), *n*>20 vesicles from five independent experiments.

As has been previously reported (Takahashi *et al.*, 2013), protein abundance can modify the contours of vesicles, allowing the formation of soft GVPMs with a non-spherical shape (Fig. 6A). Moreover, GVPMs containing proteins and lipids exhibited a higher RGM than GUVs formed with the same lipid mixture (Fig. 6B), indicating that proteins tend to limit the packing of the membrane.

To compare local membrane order at the surface of these GUVs and GMPVs, the emission signals of fluorescently labelled vesicles were acquired on a tangential plane corresponding to membrane surfaces of 100–350 µm^2^ (Fig. 6A), and the distribution of RGR values was determined (Fig. 6C). The distribution for PM lipid GUVs was centred towards low values (from 0.6–1.1; Fig. 6C), consistent with the high membrane order of these GUVs (Fig. 6B). In contrast, the RGR value of GVPMs exhibited a more peaked distribution shifted towards higher RGR values (from 1.1–1.4; Fig. 6C), suggesting that the presence of protein induced a shift towards fewer ordered domains. To investigate a possible concomitant adjustment of the spatial organization of ordered domains, a granulometric analysis was performed by focusing on ROIs that exhibit an RGR value in the first quartile of values, in order to eliminate any density effects. We measured an ordered domain size of 0.342 µm^2^ for GVPMs (Fig. 6D); interestingly this was similar to the value for BY-2 cells (i.e. 0.350 µm^2^, Fig. 4D). Moreover, this size tended to be lower than the size of GUVs composed of a PM lipid mixture (Fig. 6D), suggesting a negative impact of the presence of protein on both the quantity and size of ordered domains at our scale of observation. To better understand the dissolution of the ordered domains in native PM conditions, ratiometric images were further segmented to only take into account ROIs exhibiting red/green ratios below a RGR value of 1.2, which corresponds to one population of ordered domains (Grosjean *et al.*, 2015). This ordered domain fraction was calculated, and a significant decrease was observed for GVPMs in comparison to GUVs (Supplementary Fig. S9A), confirming that the reduction in ordered domain abundance was predominantly involved in the low membrane order reported for GVPMs (Fig. 6B). To determine the lateral organization of these ordered domains within PM vesicles, we analysed their degree of clustering. The group size of ordered domains revealed a protein-dependent decrease, with the presence of ordered domains exhibiting a mean size of ~0.8 µm^2^ in GUVs composed of only PM lipids and ~0.6 µm^2^ in GVPMs (Supplementary Fig. S9B). We observed a linear correlation between the size of ordered domains characterizing GUVs composed of different lipid mixtures and the membrane order (Supplementary Fig. S9C). This correlation disappeared in GVPMs (Supplementary Fig. S9C), suggesting that the presence of proteins decreases the number of ordered domains, but concomitantly induces another mechanism that can reduce the propensity of ordered domains to lie within clusters.

Taken together, these results support the ability of PM lipids and proteins to finely govern PM order, and to subsequently control the tenuous organization of the PM.

## Discussion

### Lipids and proteins can account for the structuring of plant PM ordered domains

In this study we determined a high level of membrane order for GUVs mimicking the native composition of tobacco BY-2 PM lipids. This included 38% GIPCs, 8% free phytosterols, and 10% conjugated phytosterols, which were associated with a large and continuous distribution of individual levels of membrane order exhibited by different membrane regions of these vesicles. One possible explanation is that the ‘lipid raft’ model is also applicable to plants. This model places the local interactions between lipid species (Ramstedt and Slotte, 2006; Quinn, 2010) as the first level of membrane organization (Lingwood and Simons, 2010). Consistent with this hypothesis, plant-specific conjugated sterols display a striking ability to induce ordered domain formation in the membrane that acts in synergy with the similar ability of free phytosterols (Grosjean *et al.*, 2015). Indeed, phytosterols increase membrane stiffness and the inclusion of other lipids, depending on their structure and shape (Shahedi *et al.*, 2006), resulting in macromolecular assemblies and lipid bilayer ordering (Mannock *et al.*, 2015; Shaghaghi *et al.*, 2016). Furthermore, formation of sterol-dependent membrane domains is modified by the addition of GIPCs (Grosjean *et al.*, 2015). This adds weight to a model in which the combination of multiple molecular species of phytosphingolipids and phytosterols present in living tobacco cell PMs may induce membrane ordering and enhance the formation of various ordered domains. The high diversity of plant PM lipids could then increase opportunities for local interactions with different intensities, and consequently bring about heterogeneity at the local membrane compaction level, providing an explanation for the wide range of RGR values measured for GUVs consisting of tobacco PM lipid components. RGR values, which corresponded to average ratios of the smallest area optically possible to analyse (a 300 × 300 nm ROI), might reflect the mean membrane order of sub-population domains co-existing in varying proportions within these areas. Such a complex model assumes a multitude of nanodomains with levels of order between (rather than strictly corresponding to) the Lo and Ld phases (Bagatolli *et al.*, 2010). In agreement with this new degree of complexity of PM organization, the diversity of mammalian lipid mixtures has been speculated to favour the formation of ultra-nanodomains (Pathak and London, 2015). In accordance with this, the formation of distinct nanodomains has been simulated in the outer leaflet of an idealized mammalian PM consisting of a complex mixture of 63 different lipid species (Ingólfsson *et al.*, 2014).

To investigate the influence of protein–lipid interactions on PM lateral organization, we characterized GVPMs produced from tobacco BY-2 cell PMs using a novel procedure. These GVPMs, which contain hundreds of integral membrane proteins and lipids (corresponding to native PM amounts), provide a very interesting system, and yet they are rarely used due to the difficulty in obtaining them. In contrast to GPMVs, which are isolated after chemical treatment of animal cells that induces formation of detachable PM blebs (Levental and Levental, 2015), the electrofusion procedure utilized here allows the initial characterization of isolated PM organization in a native resting state. In agreement with our observations on tobacco BY-2 cells, GPMVs from animal cells have shown a clear segregation into Lo/Ld phases, with a lateral distribution that depends on the overall protein content (Baumgart *et al.*, 2007). Using a cell-swelling procedure to isolate PM spheres, the cholera toxin B subunit-dependent formation of ordered domains has also previously been reported (Lingwood *et al.*, 2008). Here, by comparing the GUVs of PM lipids and the GVPMs, we further demonstrated the involvement of PM proteins in the modulation (especially loosening) of plant PM order. Indeed, when proteins were positioned between lipid molecules, they increased the membrane line tension and modified the mean size of the ordered domains in the tobacco PM, which has also been reported for lung surfactant monolayers (Dhar *et al.*, 2012). Peptides with a short hydrophobic transmembrane domain accordingly decrease the affinity of sterols for neighbouring phospholipids (Nyström *et al.*, 2010; Nyholm *et al.*, 2011; Ijäs *et al.*, 2013), suggesting that proteins could modify membrane lateral organization by excluding certain lipids from the closest surrounding bilayer. Moreover, lipids in direct interaction with proteins result in areas that are much less compact in the immediate vicinity of the proteins (Brannigan and Brown, 2006), since protein–lipid interactions depend on the size and the charge of chemical groups present at the protein surface (Honig *et al.*, 1986). The anchoring of transmembrane proteins into ordered domains is also hypothesized to redistribute ordered domains (Epand *et al.*, 2004; Epand, 2008).

We are thus able to propose a final model in which lipid–lipid interactions may control the formation of ordered domains at the plant PM surface (whereas protein–lipid and/or protein–protein interactions contribute to drive the whole-membrane organization). With this model in mind, we noticed a remarkable limitation in the size of ordered domains within GVPMs, which could undoubtedly be attributed to the presence of proteins. Indeed, GVPMs and living cells both exhibit similar small ordered domains, and this small size originates from the ability of proteins to emulsify ordered domains (Bhatia *et al.*, 2016). This PM organization should be based on hydrogen bonds and van der Waals interactions operating on a time scale representative of what we observed, and should show, in a coherent manner, an insensitivity to protein kinase inhibitors.

### The cell wall–PM–cytoskeleton continuum is not a common hallmark of domain assembly

Besides local membrane composition, domain formation could also be related to a limited lateral diffusion of PM components. The cortical cytoskeleton in particular has been proposed to generate barriers that constrain movement in the membrane, as revealed by the restricted diffusion of PM proteins in certain membrane compartments (Kusumi *et al.*, 2005; Umemura *et al.*, 2008). Furthermore, the hindered diffusion of phospholipids and sphingolipids was similarly abolished in actin cytoskeleton-free cell-derived GPMVs, as measured using super-resolution stimulated emission depletion microscopy combined with fluorescence correlation spectroscopy (Schneider *et al.*, 2017). These observations support the ‘picket fence’ model, in which transmembrane proteins, like pickets, are anchored to and lined up along a ‘fence’ of cytoskeletal proteins surrounding the confinement zones (Kusumi *et al.*, 2005). In plants, single-particle tracking analysis has recently revealed that cytoskeleton integrity, especially microtubules, restricts the lateral mobility of plant innate immunity proteins such as AtHIR1 at the PM surface of Arabidopsis cells (Lv *et al.*, 2017). However, quantifying protein diffusion using fluorescence recovery after photobleaching experiments has previously demonstrated that the cytoskeleton is not responsible for the relative immobility of plant PM proteins (Martinière *et al.*, 2012). This apparent contradiction could be explained by different sensitivities to microtubule disturbance depending on the particular protein that is observed (Szymanski *et al.*, 2015). This shows that multiple types of membrane domains (which are specifically enriched in one or the other of these proteins) co-exist at the same time in plants (Jarsch *et al.*, 2014). Some of these domains may reflect the formation of specific areas enriched in PM components that are temporarily trapped by the cytoskeleton network within distinct PM sub-regions, as has been proposed in animal cells (Murase *et al.*, 2004). Among these domains, the composition of DRM-associated domains has been shown to be under the control of the cytoskeleton network, since microtubules regulate the dynamics of DRM-marker proteins such as Arabidopsis Flot1 (Li *et al.*, 2012) or Arabidopsis remorin (Szymanski *et al.*, 2015).

In plants, these domains may co-exist with other domains whose formation has been proposed to be directly subject to lipid and protein composition (Urbanus and Ott, 2012), and that have been revealed in our present study. Indeed, by comparing the membrane organization of living tobacco cells affected (or not) in the structure of their actin and/or tubulin networks, we have shown that the cytoskeleton does not impact on the short-term regulation of the distribution of PM-ordered domains in tobacco. To the best of our knowledge, only one previous study has reported that alteration of either actin content or its association with the PM affects the physical properties of the PM in animal cells, e.g. disruption of the cortical cytoskeleton coinciding with specific limitations of the ordered domain fraction (Dinic *et al.*, 2013). However, this actin-dependent formation of ordered domains was demonstrated to occur at 37 °C. At this temperature, lipid–lipid interactions reduce the formation of ordered domains in GPMVs (Baumgart *et al.*, 2007), giving prominence to interactions between the membrane and intracellular filaments. In line with this, it has been proposed that ordered domains are too small to be optically detected at high temperature, regardless of the lipid composition (Pathak and London, 2015). In order to be able to compare our different systems, we performed all of our experiments at room temperature (24 °C), as it is convenient for cultivating plant cells. At this temperature, we observed that the presence and characteristics of ordered domains essentially depended on the lipid and protein mixtures present within the membrane.

In this study, we attempted to identify all the cellular elements underlying the heterogeneity of PM biophysical properties in plant cells at a specific time, using as a system a tobacco cell PM that had already been synthesized and was in a steady state. The results we obtained in characterizing the membrane order of protoplasts indicated that the cell wall does not play a key role in the maintenance of membrane order, or in the spatial organisation of ordered domains in resting tobacco cells. Even though direct interactions between PM proteins and cell wall components are known to constrain the lateral diffusion of PM proteins (Martinière and Runions, 2013), no involvement of cell wall organization in PM packing has been reported to date, and our results tend to exclude such a hypothesis. Furthermore, the absence of an effect of short-term cytoskeleton disorganization also questions the relevance of the ‘picket fence’ hypothesis in plant cells. Instead, our data suggest that lipids and proteins are the main determinants involved in the organization of membrane order in tobacco BY-2 cells. In agreement with our model, studies on the brassinosteroid binding receptor and its co-receptor have shown that the formation of nanoclusters within the PM of Arabidopsis seedlings is mainly subject to biophysical restraints, whereas cytoskeleton disruption does not have any effect on this parameter (Hutten *et al.*, 2017). The complex mechanisms by which the PM is synthesized and renewed might be a way to control the targeting of the different membrane components (Zarský *et al*., 2009; Kleine-Vehn *et al.*, 2011), and consequently to control the interactions between neighbouring lipids and proteins. In accordance with this, modifications of PM composition in an Arabidopsis mutant have been reported to alter membrane order at the resting state (Sena *et al.*, 2017). According to the limited data available, such a metabolic turnover of membrane components might occur over a time frame of several hours (Shi *et al.*, 2008; Stanislas *et al.*, 2009; Chalbi *et al.*, 2015), which far exceeds our experimental conditions.

### Ordered domains are part of PM lateral heterogeneity

Our results highlight the possible co-existence of distinct domains: the first corresponds to PM domains, in which proteins or oligomers of proteic complexes exhibit restricted lateral diffusion, and the second corresponds to ordered domains with a sterol-enriched composition. Thus, microscale domains of restricted diffusion proteins might be distinct from nanoscale ordered domains (observed here using a lipid packing-sensitive probe), since the mechanisms responsible for their formation are most probably different. One hypothesis in animals posits that PM organization synthesizes a multiscale organization of: (i) plasma membrane compartments (i.e. microscale-sized domains) partitioned through membrane component entrapment that depends on the actin-based cytoskeleton; (ii) lipid raft domains (2–20 nm) created via sphingolipid–sterol interactions; and (iii) protein complexes (3–10 nm) composed of dimer/oligomer assemblies (Nicolson, 2014). In addition to the cytoskeleton-dependent formation of PM sub-regions with restricted lateral diffusion of their components mentioned above, immuno-electron microscopy experiments in plants have revealed that the lipid PIP2 is partitioned into 25-nm clusters (Furt *et al.*, 2010). Furthermore, the protein remorin is similarly aggregated into 70-nm domains in the cytosolic leaflet of tobacco leaf PMs (Raffaele *et al.*, 2009). These two lines of evidence argue in favour of the existence of integrated levels of PM organization in plants similar to that found in animals.

In the PM of tobacco cells in a resting state, domains of restricted lateral diffusion may thus co-exist with ordered domains that are compatible with the size investigated here, and for which we have demonstrated that lipid–lipid and/or protein–lipid interactions are the major driving forces of their formation. Interestingly, both of these domains have recently been implicated in plant stress responses, and notably in plant immunity (Bucherl *et al.*, 2017). Future work should aim to unravel the mechanisms used to regulate the number of ordered domains, the dynamics of which are known to be involved in the early stages of defense responses (Liu *et al.*, 2009; Gerbeau-Pissot *et al.*, 2014; Sandor *et al.*, 2016).

## Supplementary data

Supplementary data are available at *JXB* online.

Fig. S1. Effect of staurosporine on RGM of control and cryptogein-elicited BY-2 cells.

Fig. S2. Influence of cytoskeletal-active compounds on the viability of tobacco cells.

Fig. S3. Influence of the cytoskeleton on the membrane order of BY-2 PMs measured by spectrofluorimetry.

Fig. S4. Comparison of the distribution of RGR values between control cells and cells treated with cytoskeletal-active compounds.

Fig. S5. Influence of cell wall–PM connections on the level of tobacco cell PM order.

Fig. S6. Lipid composition of PMs isolated from tobacco suspension cells.

Fig. S7. Effect of different lipid compositions on the membrane order of giant vesicles.

Fig. S8. Influence of swelling-solution composition on GVPM size.

Fig. S9. Formation of large ordered domains in giant vesicles made up of tobacco PM lipids and proteins.

Supplementary Figures

## Abbreviations:

DOPC: 1,2- dioleoyl -*sn*-glycero-3- phosphocholine
DPPC: 1,2- dipalmitoyl -*sn*-glycero-3- phosphocholine
GUV: giant unilamellar vesicle
GVPM: giant vesicles of native plasma membrane
GPMV: giant plasma membrane vesicles
RGM: red-to-green ratio of membrane fluorescence
RGR: red-to-green ratio for a specific region of interest.
