# Supplementary material for: Interactions between lipids and proteins are critical for organization of plasma membrane-ordered domains in tobacco BY-2 cells
Source: J Exp Bot. 2018 May 2;69(15):3545–57. doi: 10.1093/jxb/ery152 (PMC6022670; doi:10.1093/jxb/ery152)
Supplement: Supplementary Figures [file ery152_suppl_supplementary_figures.pdf]

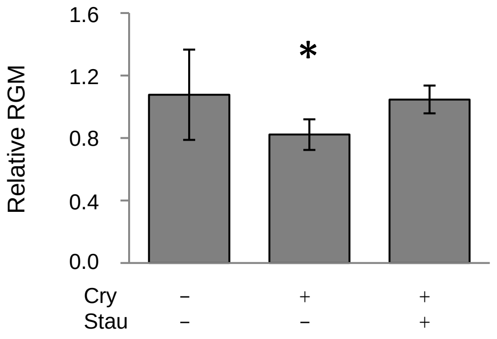

Supplementary Figure S1. Staurosporine effect on RGM of control and cryptogein-elicited BY-2 cells. The red/green ratio of the PM (RGM) was measured by spectrofluorimetry after incubation of cultured tobacco BY-2 cells with 50 nM cryptogein (Cry), an elicitor of plant defense reactions, and with or without 2.5 µM staurosporine (Stau), a protein kinase inhibitor. Mean values ± SD, n>6 independent experiments. The asterisk indicates a significant difference (p-value<0.05).

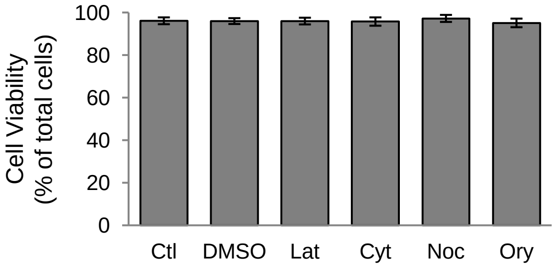

Supplementary Fig. S2. Influence of cytoskeletal drugs on the viability of tobacco cells. Cytoskeletal drugs (latrunculin B, Lat, 10 µM; cytochalasin D, Cyt, 50 µM; nocodazole, Noc, 20 µM; and oryzalin, Ory, 10 µM) were added to BY-2 cells. After one hour of incubation, the effect of cytoskeletal drugs on cell viability was quantified by dual fluorescence staining using propidium iodide (PI, 20 µg.ml^{-1}, dead cells) and fluorescein diacetate (FDA, 0.001%, living cell). After 2 min of labelling, tobacco cells were examined with a Zeiss Axiophot microscope, equipped with a 40x objective lens (NA = 0.75) and epifluorescence (filters used for PI: BP546±12/FT580/LP4 590 and FDA: BP450-490/FT510/LP520). More than 300 cells were counted for each experiment and the viability percentage was evaluated. Mean values ± SD, n>4 independent experiments.

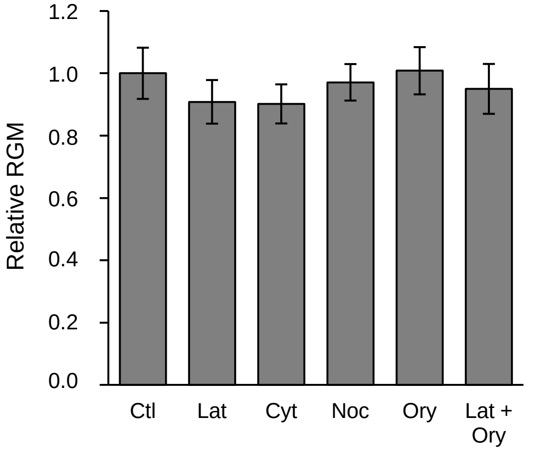

Supplementary Fig. S3. Influence of cytoskeleton on the membrane order of BY-2 PMs measured by spectrofluorimetry. Cytoskeletal drugs (latrunculin B, Lat, 10 µM; cytochalasin D, Cyt, 50 µM; nocodazole, Noc, 20 µM; and oryzalin, Ory, 10 µM) were added to BY-2 cells. After a 1-h incubation, the membrane order of individual cells was quantified by spectrofluorimetry and RGM was reported relative to the value obtained without pharmacological treatments (control, Ctl). Mean values ± SD, n>5 independent experiments.

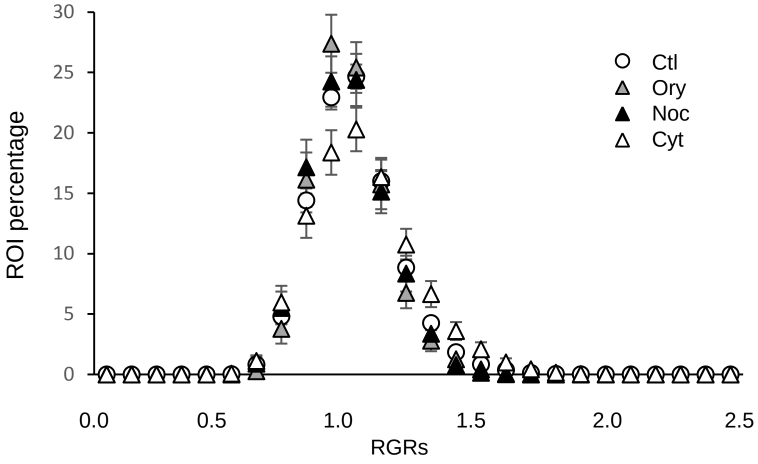

Supplementary Fig. S4. Comparison of the distribution of RGR values between control and treated cells. RGR values are shown in control (Ctl, circles) and treated (triangles: grey, Ory, oryzalin, 10 µM; black, Noc, nocodazole, 20 µM; white, Cyt, cytochalasin D, 50 µM) cells. The x-axis represents the class of RGR values; only the maximal value of each class is reported on the graph. The y-axis represents the percentage of each class of ROI values. Mean values ± SEM; n>90 cells from 5 independent experiments.

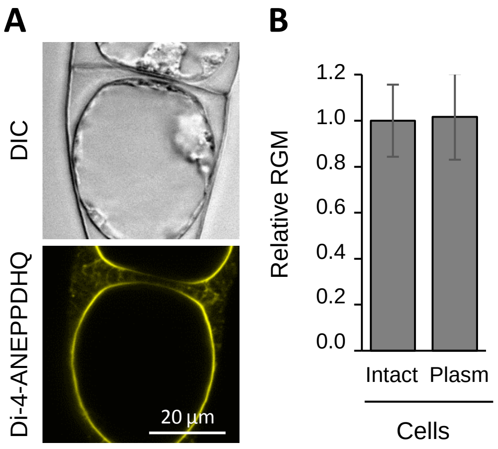

Supplementary Fig. S5. Influence of cell wall-PM connections on the level of tobacco cell PM order. PM-cell wall disconnection was obtained by incubating tobacco cells in a hyperosmotic condition induced by a 400 mM mannitol concentration gradient. A, Cell morphology (differential interference contrast, DIC) and plasma membrane localization (fluorescence imaging after di-4-ANEPPDHQ labelling, di-4-ANEPPDHQ) observations. B, Analysis of the effect of PM-cell wall disconnection on membrane order, by measuring the RGM of tobacco cell PMs detached from the cell wall due to severe plasmolysis. Mean values ± SD, n>81 cells.

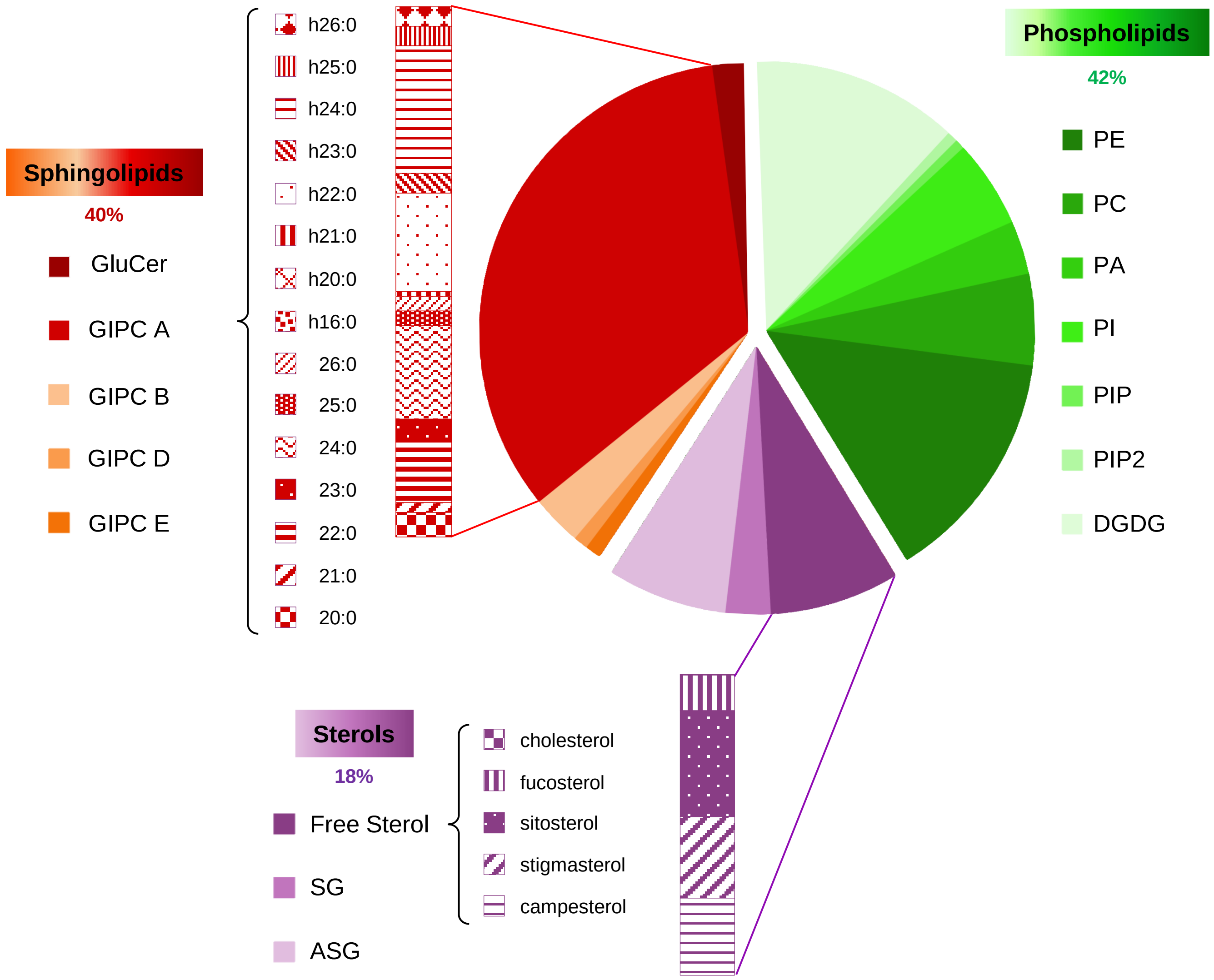

Supplementary Fig. S6. Lipid composition of PM isolated from tobacco suspension cells. Lipid content of tobacco PMs was determined by quantifying lipid classes that were purified using different protocols. The three main lipid classes, namely phospholipids (green colours), sterols (purple colours), and sphingolipids (red colours), are represented as the percent weight of all lipids (represented by the whole circle). Diversity is illustrated for classes (shades of colours) and sub-classes for two representative examples (stacked bars). The major lipid molecules were detailed, *i.e.* sphingolipids (GluCer and GIPCs), sterols (free sterols, SG, and ASG) and glycerolipids (PE, PC, PA, PI, PI4P, PI4,5P2, and DGDG). Data are from three independent experiments. Abbreviations are as follows: glucosylceramide (GluCer), glycosyl inositol phosphorylceramides (GIPC), steryl glycosides (SG), acyl steryl glycosides (ASG), phosphatidylethanolamine (PE), phosphatidylcholine (PC), phosphatidic acid (PA), phosphatidylinositol (PI), phosphatidylinositol 4-phosphate (PIP), phosphatidylinositol 4,5-bisphosphate (PIP2), digalactosyldiacylglycerol (DGDG).

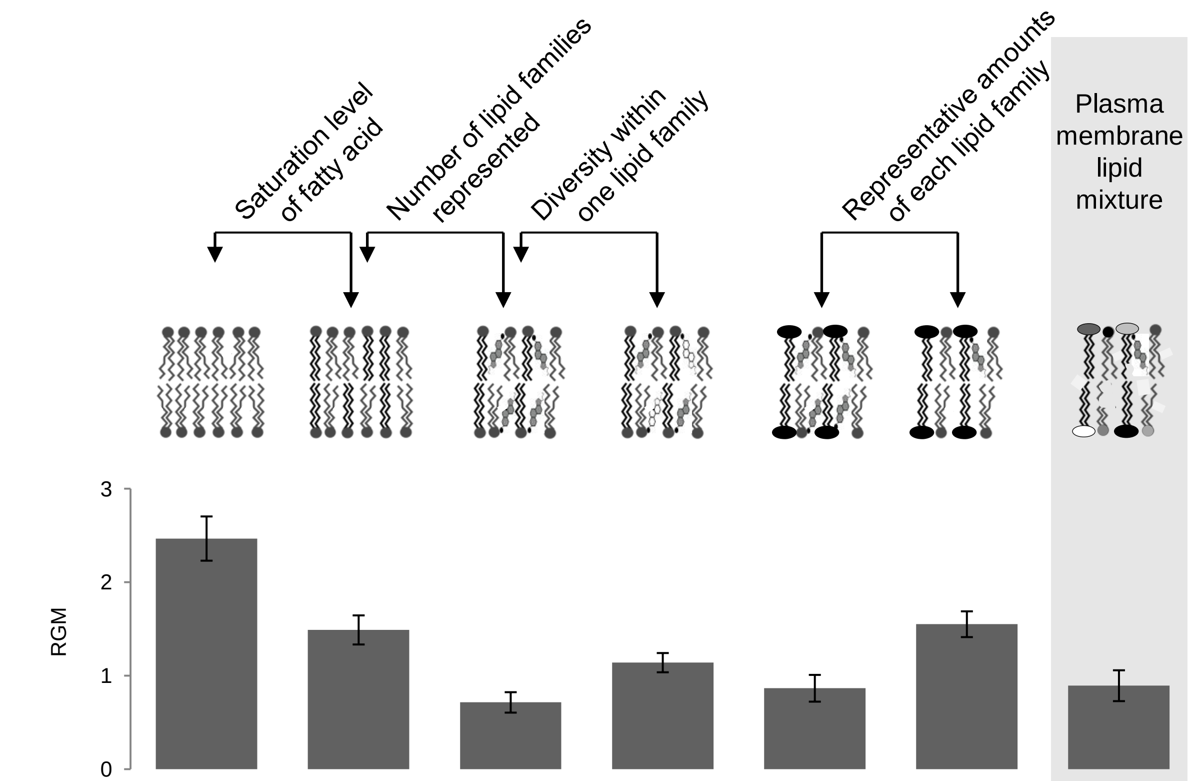

Phospholipids:	DOPC	DOPC/DPPC	DOPC/DPPC	DOPC/DPPC	DOPC	DOPC	Tobacco
Sphingolipids:	-	-	-	-	SM	SM	Tobacco
Sterols:	-	-	Cholesterol	Phytosterols	Cholesterol	Cholesterol	Tobacco
Molar ratio:	1	1/1	1/1/1	1/1/1	1/1/1	4.5/4.5/1	4/4/1.5

Supplementary Fig. S7. Effect of different lipid compositions on membrane order of giant vesicles. The RGM of GUVs labelled with di-4-ANEPPDHQ (3 µM, several min) was measured by confocal microscopy. GUVs were produced using different lipid mixtures for which the composition and the proportion (in molar ratio) of each lipid family are reported. Data shown are mean values ± SD, n=5 or more independent repetitions.

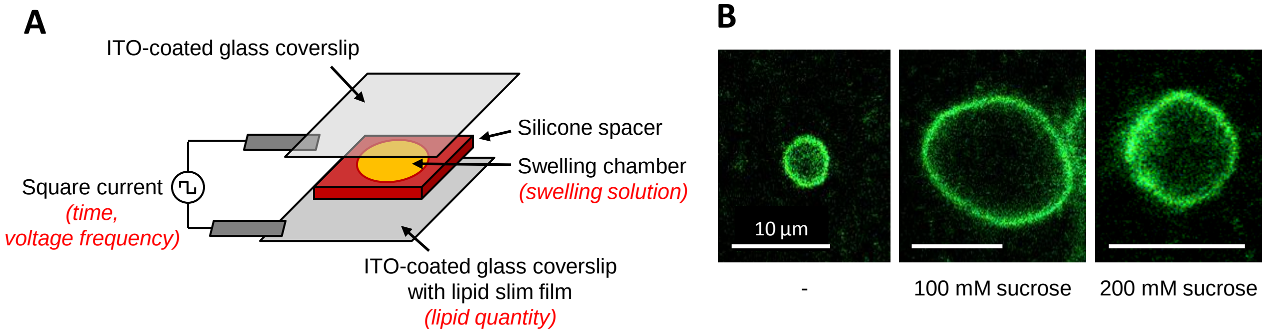

Supplementary Fig. S8. Influence of swelling solution composition on GVPM size. A, Optimized protocol for high efficiency production of giant vesicles of native tobacco PMs. PM vesicles (2 µg, 150-200 nm in diameter) were placed in the swelling chamber filled with 200 µl of a solution of HEPES (25 mM), NaCl (10 mM) and various concentrations of sucrose (0, 100 or 200 mM). Note that all parameters in which the effect has been tested are reported in red. The best conditions were determined to be a voltage of 3.5 V at 10 Hz and a temperature of 40°C. B, Microscopic evaluation of the size of di-4-ANEPPDHQ-labelled GVPMs, after 2 h of swelling. Representative GVPMs are shown for each condition.

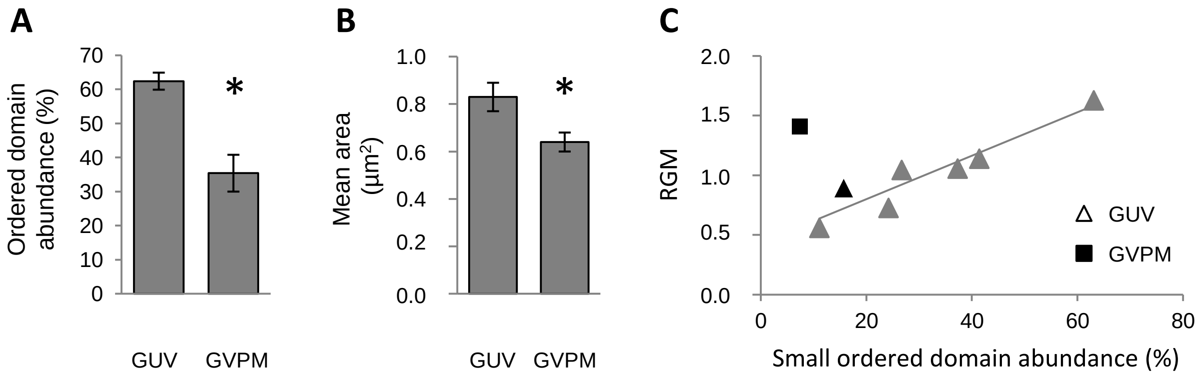

Supplementary Fig. S9. Formation of large ordered domains in giant vesicles made up of tobacco PM lipids and proteins. A, Proportion of ordered domains. ROIs exhibiting an RGR value below a threshold of 1.2 were counted as a giant vesicle composed of either PM lipids (GUV) or PM lipids and proteins (GVPM). B, The size of ordered domains was measured as the mean area of groups of pixels corresponding to ROIs exhibiting an RGR value below threshold (<1.2, highlighting the lowest RGR values). These were compared between giant vesicles composed of either PM lipids (GUV) or PM lipids and proteins (GVPM). C, The size of areas grouping ROIs exhibiting an RGR value below a threshold of 1.2 was evaluated for different GUV mixtures (triangles) and one GVPM (square). The relative abundance of isolated ROIs (small ordered domains) is reported according to the level of membrane order (RGM). Vesicles composed of native PMs are represented in black. Data shown are mean values, n=21-38 GUVs.
